# Fluorimetric Analysis of Five Amino Acids in Chocolate: Development and Validation

**DOI:** 10.3390/molecules26144325

**Published:** 2021-07-16

**Authors:** Maria S. Synaridou, Vasilis Tsamis, Georgia Sidiropoulou, Constantinos K. Zacharis, Irene Panderi, Catherine K. Markopoulou

**Affiliations:** 1 Laboratory of Pharmaceutical Analysis, Department of Pharmaceutical Technology, School of Pharmacy, Aristotle University of Thessaloniki, 54124 Thessaloniki, Greece; msynarid@pharm.auth.gr (M.S.S.); vasilistsm@gmail.com (V.T.); sgeorgiae@gmail.com (G.S.); czacharis@pharm.auth.gr (C.K.Z.); 2 Laboratory of Pharmaceutical Analysis, Department of Pharmaceutical Analysis, Faculty of Pharmacy, National and Kapodistrian Universityof Athens, Panepistimiopolis, 15771 Athens, Greece; ipanderi@pharm.uoa.gr

**Keywords:** amino acids, chocolate, derivatization, HPLC, fluorescence

## Abstract

Amino acids present ergogenic action, helping to increase, protect, and restore the muscular system of young athletes. Moreover, the encapsulation of five relevant amino acids in chocolate pellet form will appeal to them, facilitating their daily consumption. A reliable HPLC fluorimetric method was developed to detect and quantitatively determine L-Leucine, L-Isoleucine, L-Histidine, L-Valine, and β-Alanine in chocolate using aniline as an internal standard. Experimental design methodology was used to investigate and optimize the clean-up procedure of the samples. Therefore, three extraction techniques (solid-phase extraction (by two different SPE cartridges) and liquid–solid extraction (LSE)) were compared and evaluated. The LOQ values in chocolate varied from 24 to 118 ng/g (recovery 89.7–95.6%, %RSD < 2.5). Amino acids were pre-column derivatized with *o*-phthalaldehyde (OPA), while derivatization parameters were thoroughly investigated by experimental design methodology. The analysis was performed by HPLC-fluorescence (emission: λ = 455 nm, excitation: λ = 340 nm) method using a C_18_ column and a mixture of phosphate buffer (pH = 2.8; 20 mM)-methanol as a mobile phase in gradient elution. The method was validated (r^2^ > 0.999, %RSD < 2, LOD: 10 ng mL^−1^ for histidine and leucine, 2 ng mL^−1^ for alanine and valine, and 4 ng mL^−1^ for Isoleucine) according to the International Conference on Harmonization guidelines.

## 1. Introduction

Dietary supplements are constantly gaining supporters, particularly in the field of sports. These supplements are concentrated sources of nutrients with great nutritional value; they are intended to supplement normal diets, according to each person’s needs. There are many types of food supplements on the market, such as tablets, pastilles, ampoules containing liquids, and powders, designed to be taken in measured, small-unit quantities. Alternatively, they are usually consumed with nutritious food, such as cereal bars or milk drinks [1]. Chocolate is a food product consumed worldwide, by different populations, due to its desirable sensory characteristics [2]. Dark chocolate, in particular, with its high cacao content, is one of the most promising functional foods, owing to its high levels of bioactive compounds, including flavonoids and phenolic acids [3].

Amino acids (AAs) are basic dietary supplements for those who exercise. They are also referred to as the building blocks of life due to their important roles in ribosomal protein synthesis. Protein AAs can be divided into two categories: those that cannot be synthesized by the organism (essential), and must be supplied from external sources, and non–essential amino acids that are synthesized by the organism [4].

The use of dietary supplements is determined by the amino acid content. For example, formulations containing branched chain AAs (consisting of leucine, isoleucine, and valine 2: 1: 1) are among the most widely used by athletes as they contribute to muscle recovery after training.

The scientific community has conducted a lot of research in this field, analyzing amino acids in various matrices, such as environmental samples [5], food [6,7], plants [8], or herbal raw material [9] and biological fluids [10,11]. Particular emphasis has been placed on the determination of amino acids in nutritional substrates and the purity of the sample [12].

The extraction of protein-bound amino acids usually precedes the hydrolysis stage, which is a complex process [13,14] followed by a clean-up step. For most sample matrices, solid-phase extraction (SPE) is optional, though it can greatly improve the quality of a sample by cleaning and concentrating the overall analytical outcomes [15]. In AA analysis, ion-exchange SPE is typically employed. Being zwitterionic, both cation and anion exchange SPE can be used to selectively retain amino acids. In contrast, cartridges with C_18_ are not suggested since it was reported that they had poor retention of the underivatized polar amino acids and low selectivity [16,17,18].

A variety of analytical techniques have been reported in literature regarding the determination of AAs, including capillary electrophoresis (CE) [19], thin layer chromatography (TLC) [20], liquid chromatography (HPLC) [21], or gas chromatography (GC). In most cases, HPLC is coupled with a fluorimetric (FLD) [22] or a mass spectrometric (MS) detector [13,23,24].

In general, the analysis of low molecular-weight amino acids is considered to be a challenging analytical task due to their polar nature, small size, and their relative low ability to absorb ultraviolet radiation (lack of chromophore groups). The derivatization of such molecules with appropriate reagents often helps to effectively overcome these obstacles. *O*-phthalaldehyde (OPA) is one of the most widely used reagents for the derivatization of compounds with a primary amino group [25].

The present study initially aimed to incorporate five amino acids (L-Leucine, L-Isoleucine, L-Histidine, L-Valine, and β-Alanine) in dark chocolate (pellet form) in order to prepare a complete dietary supplement. The selection of amino acids and their composition for young athletes was based on their properties to reduce fatigue (before training), and to rebuild their muscles. Thus, since the daily intake of AAs is considered essential, their formulation into dark chocolate pellets facilitates their oral intake.

According to the declaration of the European Parliament Directive (2002/46 / EC) on food supplements (Article 8–9), the values of nutrients or substances with nutritional or physiological effects contained in the product must be indicated on the label. In addition, their average values based on the product analysis, by the manufacturer, must be reported. Thus, following the process, a reliable analytical method was developed for the quantitative determination of the AAs in dark chocolate to test the suitability of the proposed formulation. Due to the complexity of the substrate, two different extraction techniques, a solid-phase extraction (SPE) using two different cartridges (NH_2_, Diol) and a liquid–solid extraction (LSE) were studied, compared, and evaluated. In order to increase the sensitivity of the method, amino acids were derivatized (pre-column) with OPA, separated by HPLC and determined by fluorescence.

The proposed method, due to its efficiency, reliability and ease of use, has a dual field of application in both routing and trace analysis.

## 2. Results

### 2.1. Optimization of the HPLC Conditions

Focusing on finding and applying a reliable chromatographic method for the determination of the amino acids, the optimization stage is considered essential. The retention time and characteristics of their chromatographic peaks (e.g., resolution and tailing factor) are directly influenced by parameters, such as the kind of the solvent or the concentration of the buffers, the flow rate, column type, and the temperature.

The biggest problem for the selection of the optimum stationary phase was the low separation of alanine and valine and the coelution of leucine and isoleucine (isomers). Since the problem was not solved using any conventional RP column, the short C_18_ (15 cm × 4.6 mm, 5.0 μm, Supelco, Bellefonte, United States) analytical column was replaced by a C_18_ DB (25 cm × 4.6 mm, 5.0 μm, Supelco, Bellefonte, PA, USA) analytical column providing better resolutions. As far as the column temperature is concerned, the increase in temperature implies a reduction in elution time, and as a result, the incomplete separation of leucine and isoleucine. Thus, among the three values studied (25, 30, and 40 °C), 25 °C was chosen as the ideal.

By using the DB analytical column, the isocratic elution did not improve the separation of the analytes; thus, a binary gradient elution was selected. Various mixtures of water with or without buffers (mobile phase A) and organic modifiers, including acetonitrile (ACN) and/or methanol (MeOH) (mobile phase B), were tested. Initially, in order to find out the most suitable mobile phase composition, a wide gradient elution system was employed (mobile phase B: 20 to 80%). The procedure was repeated in triplicate using methanol 100%, acetonitrile 100%, or methanol/acetonitrile mixture at a ratio 1:1 *v*/*v* as phase B. Under these conditions, it was observed that MeOH (as mobile phase B) and H_2_O (as mobile phase A) gave sharper peaks and, hence, were selected. Thereafter, for the final configuration of the gradient elution program, emphasis was placed on the separation of the two isomers considering their behavior data from respective isocratic mobile phases.

For further improvement of the peaks’ widths and resolution, two additional factors were examined: (a) pH adjustment using an aqueous buffer and (b) the buffer concentration. Knowing that the isoelectric point of the amino acids is about 6.0 and the pKa of their acidic group 2.3, the water was replaced with a sodium dihydrogen phosphate buffer, and studied at 2.8, 5.0, 6.0, and 7.0 pH values. The value of 2.8 was chosen as the most appropriate since the separation of the analytes improved with the reduction of the pH value. Taking into account that the high concentration of phosphate buffer, during gradient elution, should cause precipitation of salt and high back pressure in the system [22,26], three relatively low levels of buffer concentrations (10, 20, and 30 mM) were also studied. Since there were no significant improvements in the chromatographic behavior of the analytes, 20 mM was considered the ideal value. The flow rate was chosen to be low enough (0.6 and 0.7 mL min^−1^) to achieve better peak resolutions and low back pressures. Results of the system suitability parameters are depicted in Table 1.

### 2.2. Optimization of the OPA-Amino Acids Derivatization Conditions

Critical parameters affecting the amino acid derivatization, such as the time, OPA concentration, and pH of the borate buffer, were studied and optimized. Thus, with the aid of the Box-Behnken experimental design, 18 experimental combinations were performed and their results were evaluated.

**Figure 1:**
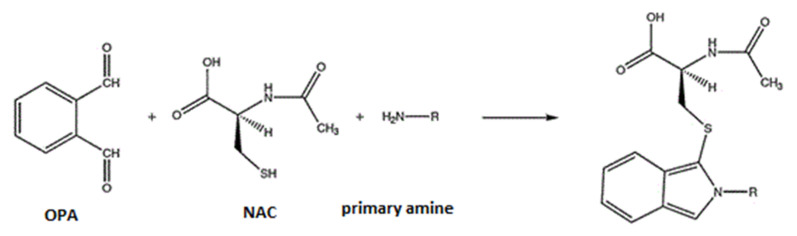
Derivatization reaction of primary amines with OPA.

The derivatization reaction of organic compounds, containing a primary amino group in their molecule, with OPA, is rapid [2,27], and leads to the formation of a fluorescence derivative due to the presence of chromophores and the rigidity of the molecule (Figure 1).

In the present experimental investigation, in order to determine the required reaction time (at ambient temperature), values ranging from 1 to 10 min were studied. As can be seen from the results of the experimental design (Supplementary Material of Figure S1), the ideal time, set as the reaction time, was 8 min.

**Figure 2:**
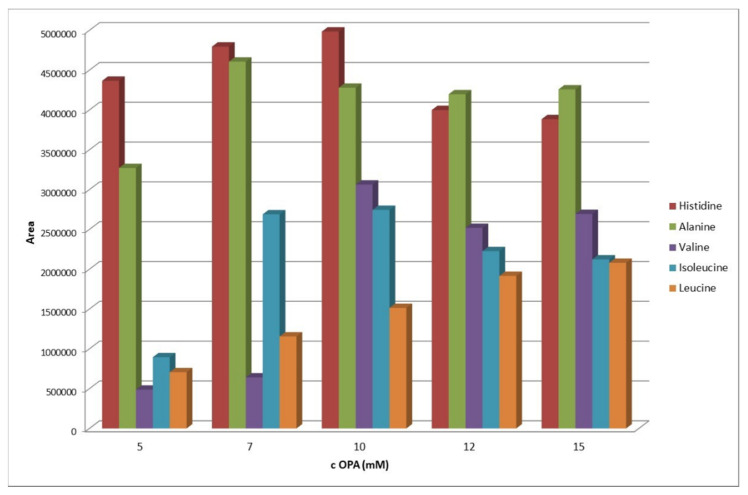
Effect of OPA concentration on the effectiveness of the reaction.

As expected, the concentration of the OPA is also one of the most critical factors that determine the effectiveness of the reaction, and is directly related to its stoichiometry. Preliminary experiments were performed using a standard solution of AAs (histidine 5 μg mL^−1^, alanine 2 μg mL^−1^, valine 10 μg mL^−1^, isoleucine 10 μg mL^−1^, leucine 20 μg mL^−1^, and aniline 10 μg mL^−1^ as IS), while the OPA concentration varied from 1 mmol L^−1^ to 20 mmol L^−1^. As shown in Figure 2, there is an almost linear increase in the signal from 5 to 10 mmol L^−1^ and a negligible decrease from that point on.

Hence, the ideal OPA concentration, for the given concentration of analytes in the sample, is 10 mmol L^−1^, which was selected for future experiments. The pKa values of the amino groups of amino acids used in the chocolate preparation range from 9 to 9.7 (Table S1—Supplementary Materials). The OPA derivatization reaction takes place under alkaline conditions to prevent ionization of the secondary amino groups. Therefore, the pH of the borate buffer was studied in the range 8.5–11.0. The ideal value indicated by the software was pH 10.5. Figure S1 (Supplementary Material) depicts the total value limits studied and the ideal conditions of the selected parameters.

### 2.3. Stability of Amino Acids-OPA Derivatives

According to the literature, a major disadvantage of OPA amines-derivatives is their short shelf life [2]. For this reason, their stability was tested at 0, 12, and 24 h. More specifically, three different concentrations of derivatized mixtures of amino acids were prepared and measured (three replications at 25 °C) by HPLC. The within days %RSD of AAs derivatives was <2.0%, which indicates their stability up to 24 h.

### 2.4. Extraction Recovery–Preliminary Experiments

Chocolate is a complex matrix and, moreover, API’s determination is difficult, especially when amino acids behave in the same way as the substrate. Thus, a purification step, which would aim at the precipitation of proteins/saturated fats, and improve the efficiency of the method, is crucial. Therefore, the appropriate experimental conditions that will promote the best recoveries should be selected after investigation.

Two extraction techniques, LSE and SPE, were used in the present study. In the case of liquid/solid extraction, acetonitrile was chosen as the protein precipitant because it offers good recovery and removes most of the impurities. However, in order to achieve the complete release of the analytes, as well as for their dissolution, the presence of water in the diluent is considered necessary. Hence, after a series of experiments, it was found that the ratio of extraction solvents with the best recovery results was H_2_O-ACN 30:70% *v*/*v*. It must be noticed that the freezing of the sample for at least 45 min after its centrifugation is one more determinant parameter for the precipitation of impurities.

LSE is carried out in a single step avoiding significant losses of the analytes (Table 2) [28]. The method displays high recoveries with good reproducibility, while its relative low sensitivity in trace analysis could be considered a drawback.

For the SPE procedure, two types of cartridges, Diol and NH_2_, were tested. In this case too, special attention must be paid to the stage of protein/impurities precipitation. Thus, the amino acid extraction from the chocolate matrix with the appropriate mixture of solvents (15 mL water-35 mL ACN) should be carried out as a preliminary step. Then, after appropriate dilutions, the sample loaded into the cartridge should contain the minimum amount of water (H_2_O-ACN, 5:95% *v*/*v*). The recovery results of the analytes, at three concentration levels/three replicates, are presented in Table 3. From a chromatographic point of view, the samples obtained from the Diol cartridge were better, as there were no additional peaks in the chromatogram (see Section 2.6.1).

### 2.5. Improvement of Trace Analysis

For the trace analysis of AAs in the chocolate substrate, the combination of the two most effective purification procedures (LSE and SPE with Diol cartridge) were used. Due to the large number of parameters that probably affect the recovery of the analytes and, consequently, the large number of experiments needed, the study was divided into two steps; one for the LSE and the other for the Diol-SPE. In both cases, the efficiency of the extraction procedure was investigated, taking into account the influence of four parameters. For the LSE technique, there were the chocolate weight, the centrifugation, freezing, and ultrasonication time (Table S2A,B–Supplementary Material), whereas for the Diol cartridge there were the diluent’s initial volume, the pH of the initial diluent, the load volume of the sample, and the elution volume (Table S3A,B–Supplementary Material). All parameters were evaluated using experimental design methodology (Design Expert 11 software) and by applying the two-level factorial mode. In each case, (two models) 16 experiments were performed, and their results were evaluated in terms of %Recovery values for each amino acid (responses). The proposed solutions for the two models were combined and determined the final conditions of the overall experiment (Table 4), which were tested at three concentration levels for each amino acid (three replications). The %Recoveries and %RSD values of the experiments revealed that the proposed method is robust and can be used for the quantitation of the amino acids in chocolate at very low concentrations (Table 5). The %Prediction Error was studied in order to evaluate the predictive capability of the experimental design models (Table 6) and was found to be < 10.7. The LOQ values in chocolate were found to be 24 ng/g for histidine and alanine, 35 ng/g for valine, 118 ng/g for isoleucine, and 59 ng/g for leucine.

### 2.6. Method Validation

#### 2.6.1. Selectivity of the Developed Chromatographic Method

**Figure 3:**
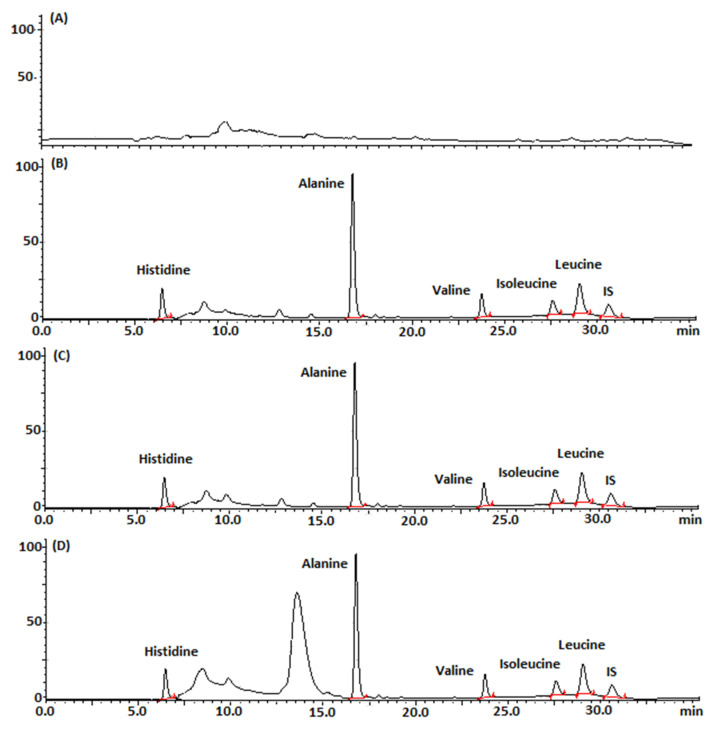
Chromatograms of the analysis of (**A**) blank chocolate sample; (**B**) spiked sample after LSE; (**C**) spiked sample after SPE-Diol; and (**D**) spiked sample after SPE-NH_2_.

Chromatograms of drug-free chocolate samples (blank) were compared with three corresponding samples spiked with histidine at 0.18 μg mL^−1^, alanine at 0.3 μg mL^−1^, valine and isoleucine both at 0.6 μg mL^−1^, leucine at 1.2 μg mL^−1^, and aniline (IS) at 3 μg mL^−1^. Both series of samples were processed using three different clean-up methods and derivatized with OPA. As can be observed in the chromatograms shown in Figure 3B–D, there are no co-eluted matrix interferences at the retention times of the analytes. Of course, in the case of the SPE with the -NH_2_ cartridge, there is an additional peak near alanine, which essentially makes its quantification difficult.

#### 2.6.2. Linearity, LODs, and LOQs

The linearity of the method was studied using five standard solutions with different concentration ranges for each amino acid (three replicates). Results of linearity regression, correlation coefficient, LOD, and LOQ for the amino acids are presented in Table 7.

#### 2.6.3. Accuracy and Precision

To evaluate accuracy, spiked placebo samples were analyzed in triplicate at three different concentration levels (0.09–5.0 μg mL^−1^ for histidine, 0.2–2.0 μg mL^−1^ for alanine, 0.3–8.0 μg mL^−1^ for valine and isoleucine, and 0.6–15.0 μg mL^−1^ for leucine. The %Recovery and %RSD values were used as evaluation criteria for each purification procedure. As indicated in Table 2; Table 3, recovery values were within the acceptable limits (98.4–100.2 for LSE, 97.8–100.6 for SPE-Diol, and 96.9–100.3 for SPE-NH_2_), whereas the %RSD values ranged from 0.01 to 0.02 for LSE, 0.46 to 1.32 for SPE-Diol, and 0.47 to 3.01 for SPE-NH_2_, respectively. However, considering that during the extraction of the AAs with the SPE-NH_2_ cartridge, an additional peak appears in the chromatogram (close to alanine), the method was rejected, while both LLE and SPE-Diol were further examined with experimental design, and their combination was used for the trace analysis of amino acids in chocolate.

To evaluate the precision of the method, repeatability and intermediate precision were examined. Therefore, a standard solution of amino acids (3.0 μg mL^−1^ for histidine, 1.0 μg mL^−1^ for alanine, 5.0 μg mL^−1^ for valine and isoleucine, and 10.0 μg mL^−1^ for leucine) was analyzed (six replicates) on the same day. The %RSD values were found to be <2%. Regarding the intermediate precision, samples in three different concentrations (0.02–5.0 μg mL^−1^ for histidine, 0.01–2.0 μg mL^−1^ for alanine, 0.01–8.0 μg mL^−1^ for valine and isoleucine, and 0.03–15.0 μg mL^−1^ for leucine) were prepared and analyzed on three consecutive days (three replicates). In all cases, %RSD values were found to be < 2% (Table S4—Supplementary).

#### 2.6.4. Robustness

The Plackett–Burman experimental design was used to evaluate method robustness [29] and to limit the number of tests needed (11 tests) (Table S5—Supplementary Table). The parameters studied were: the concentration of the buffer (sodium dihydrogen phosphate, mobile phase A) at a range of 18–20 mM, the pH values of the solution (2.7 to 2.9), the temperature of the column (from 24 to 26 °C), the initial methanol content (mobile phase B: 39 to 40% *v*/*v*), as well as the injection volume (from 19 to 21 μL). The evaluation of the experimental results was carried out based on the intensity ratios (amino acid area/internal standard), as well as on the resolution of the chromatographic peaks.

As we can see from the Pareto diagrams (Figure S2 in the Supplements), none of the parameters studied at the specific value range significantly affect (*p* < 0.05) the ratio of peak areas and the resolution. Therefore, the proposed chromatographic method is robust.

## 3. Materials and Methods

### 3.1. Chemicals, Materials and Reagents

All amino acids (L-Leucine, L-Isoleucine, L-Histidine, L-Valine, and β-Alanine) were Pharma grade (purity 98.5–101.5%) and were purchased from Merck (Darmstadt, Germany). Aniline (≥99.5%), hydrochloric acid (37% *w*/*v*), N-acetyl-cysteine (NAC) (≥99.0%), *o*-phthalaldehyde (OPA) (≥99.0%), sodium dihydrogen phosphate, boric acid, and sodium hydroxide pellets were provided by Sigma-Aldrich (Steinheim, Germany). Acetonitrile (ACN), methanol (MeOH) and water of HPLC grade were supplied by VWR chemicals (Vienna, Austria), whereas phosphoric acid, which met USP specifications, was purchased from Merck (Darmstadt, Germany). Dark chocolate (70% cacao) was obtained from local markets (Greece). SPE tubes (Diol and NH_2_) were purchased from Supelco (Sigma Aldrich, Steinheim, Germany).

Standard stock solutions of amino acids were prepared on a daily basis by dissolving the corresponding amount of each amino acid to HCl 0.1 N to obtain a 1000 μg mL^−1^ solution. Working standards were made by appropriate dilutions of the stock solutions in d. water. The OPA derivatization reagent (10 mmol L^−1^) was prepared by dissolving 5 mg of *o*-phthalaldehyde in 0.5 mL MeOH followed by dilution with 9.5 mL water and the NAC solution (10 mmol L^−1^) by dissolving 10 mg of the reagent in 0.5 mL water. Both solutions were stable at 4 °C for three working days since they were protected from light. For the preparation of the borate buffer (10 mmol L^−1^, pH 10.5), 7 mg of boric acid was dissolved in 2 mL water, and the pH of the solution was adjusted using NaOH (in pellets). Finally, the pH of the phosphate buffer (20 mmol L^−1^) was adjusted to the desired value of 2.8 by adding drops of concentrated H_3_PO_4_.

### 3.2. Instrumentation and Chromatographic Conditions

Chromatographic separations of amino acids were performed on a Shimadzu HPLC system consisting of two LC-20AD isocratic pumps, a DGU-14A degasser, an SIL-10AD autosampler and a fluorescence detector (RF-535) (Shimadzu, Tokyo, Japan). The analytical column was a reversed phase LC-C_18_ DB (250 × 4.6 mm, 5.0 μm, Supelco (Bellefonte, United States). LC-solution^®^ software version 1.25 SP4 was utilized for hardware control and data manipulation. All separations were accomplished using a binary gradient elution program. The mobile phases A and B were 20 mM phosphate buffer (pH 2.8) and methanol, respectively. Their initial ratio was 40% *v*/*v* of B and the flow rate was set at 0.7 mL min^−1^. The ratio of the mobile phase B was linearly increased to 65% in 15 min and kept constant for 9 min. Then, it was further increased to 80% in 1 min, while the flow was decreased at 0.6 mL min^−1^ and stayed constant for up to 30 min. Then, it reverted to its initial conditions (40% B and flow 0.7 mL min^−1^) in 5 min and kept constant for up to 40 min to obtain reproducible separations. The injection volume was set at 20 μL (for the analysis of the product) and 100 μL (to determine the LOD value on the chocolate substrate). The column was thermostated at 25 °C. Amino acid-OPA derivatives were detected spectrofluorimetrically at_λex/λem = 340/455 nm.

### 3.3. Derivatization Procedure

A 100 μL aliquot of standard with amino acids or sample solution was mixed with 100 μL OPA (10 mmol L^−1^), 100 μL NAC solution, and 700 μL borate buffer (10 mmol L^−1^, pH 10.5) in a 1.5 mL autosampler glass vial. After vortex mixing for 10 s, the derivatization mixture was allowed to react for 8 min at ambient temperature prior to injection into the HPLC system. Blank samples were prepared without amino acids.

### 3.4. Preparation of the Chocolate Formulation

According to international guidelines [30,31], the required daily amounts of amino acids to be consumed by a child 10–12 years old are: 42–48 mg/kg for leucine, 18–22 mg/kg for isoleucine, 23–28 mg/kg for valine, 13–17 mg/kg for histidine, and 750–1800 mg for alanine.

Taking the above into consideration, a 12-year-old child weighing an average of 40 kg should take 4.6 gr of chocolate pellets per day (divided into 2–3 doses before and after training) containing the amounts of amino acids listed in Table 8.

For the preparation of the pellets, precisely weighed quantities of amino acids corresponding to 30 formulations were transferred to a stainless-steel bowl and blended for 10 min using a Turbula T2F shaker (WAB, Muttenz, Switzerland). Then, the required amount of chocolate (slightly melted, 30–40 °C) was added and mixing was continued for another 5 min. The mixture was placed in a silicone mold and allowed to cool in a refrigerator for 1 h.

### 3.5. Sample Purification Procedures

Four different procedures for the purification of chocolate samples and the quantification of amino acids on such a substrate are described below. The latter focuses on trace analysis.

#### 3.5.1. Liquid–Solid Extraction (LSE)

A total of 500 mg of the formulation (chocolate pellets enriched with amino acids), which was previously dissolved in 10 mL H_2_O, was placed in a water bath set at 30 °C. The sample was sonicated for 10 min and stirred for another 10 min. A volume of 2 mL of the supernatant was transferred to a centrifuge tube to which 3 mL of ACN and 5 mL of water was added. The sample was centrifuged for 15 min at 4000 rpm and then was left in the freezer for 60 min. An aliquot of 1 mL of the supernatant was diluted with water to 100 mL. Derivatization (according to the procedure) and analysis in HPLC followed.

#### 3.5.2. Solid-Phase Extraction (SPE)

A solid-phase extraction method using two types of cartridges (Diol and NH_2_) was developed to clean-up the sample and extract the amino acids. More specifically, 500 mg chocolate pellets were dissolved in a mixture of 15 mL water–35 mL can, followed by sonication for 10 min (30 °C), and stirring for another 10 min. The sample was centrifuged for 15 min at 4000 rpm; 1 mL of supernatant was diluted up to 100 mL with H_2_O-ACN (5–95% *v*/*v*) (Sample 1); 1 mL of sample passed through the column (Diol and NH_2,_, respectively) after first being conditioned with 1 mL H_2_O-ACN (5–95% *v*/*v*) and 1 mL ACN. The column was then rinsed with 1 mL of ACN and the amino acids were eluted with 1 mL of water. Derivatization was performed (as mentioned) and the samples were analyzed in HPLC.

#### 3.5.3. Trace Analysis of AAs in Chocolate

A total of 850 mg of chocolate were dissolved in 5 mL of ACN-H_2_O mixture 95–5% *v*/*v*. The sample was sonicated for 20 min (30 °C) and stirred for 10 min. The sample was centrifuged for 15 min at 4000 rpm and then put into a freezer for another 45 min; 2 mL of supernatant passed through the Diol column after firstly being conditioned with 1 mL H_2_O-ACN (5–95% *v*/*v*) and 1 mL ACN. The column was then rinsed with 1 mL ACN and the amino acids were eluted with 0.3 mL water. Derivatization was performed (as mentioned) and the samples were analyzed in HPLC.

### 3.6. Validation of the Proposed Method

The proposed method was validated according to ICH and U.S. Food Drug guidelines [29,30,31,32,33]. The parameters assessed were specificity, linearity, precision, accuracy, LOD/LOQ, and robustness.

#### 3.6.1. Specificity

The retention times of the amino acids, as well as the corresponding peaks of blank and spiked placebos samples, were compared in order to investigate the ability of the proposed method to unequivocally assess the analytes.

#### 3.6.2. Linearity

For the assessment of linearity, standard solutions were prepared by appropriate dilution of a stock standard at the ranges of 0.02–5.0 μg mL^−1^ for histidine, 0.01–2.0 μg mL^−1^ for alanine, 0.01–8.0 μg mL^−1^ for valine and isoleucine, and 0.03–15.0 μg mL^−1^ for leucine (five concentrations for each analyte). Each standard was analyzed in triplicate. A linear regression line was calculated for each amino acid using the concentrations versus peak areas ratio (AAs/internal standard). The correlation coefficient (r^2^) of the regression equation was calculated to validate the linearity parameter.

#### 3.6.3. Limit of Detection (LOD) and Limit of Quantification (LOQ)

The LOD, LOQ limits [31] of the analytes were calculated based on the residual standard deviation (*σ*) and the slope (S) of the regression equation according to the following.

LOD = 3.3*σ*/S (1)

LOQ = 10*σ*/S (2)

#### 3.6.4. Precision and Accuracy

For the evaluation of the precision of the method, repeatability (intra-day) and intermediate precision (inter-day) were assessed. A standard mix solution of an intermediate concentration (0.02–5.0 μg mL^−1^ for histidine, 0.01–2.0 μg mL^−1^ for alanine, 0.01–8.0 μg mL^−1^ for valine and isoleucine and 0.03–15.0 μg mL^−1^ for leucine), as well as a series of 3 standard solutions at three different concentrations, were analyzed on the same day and on three different days, respectively. The %Relative standard deviation (%RSD) was used to assess the precision.

To assess accuracy, chocolate samples spiked with three different concentrations (0.09–5.0 μg mL^−1^ for histidine, 0.2–2.0 μg mL^−1^ for alanine, 0.3–8.0 μg mL^−1^ for valine and isoleucine, and 0.6–15.0 μg mL^−1^ for leucine) were prepared and pretreated using the three proposed techniques (LSE and SPE). Each sample was analyzed in triplicate. The %Recovery (Equation (3)) of the analyte, the %RSD and the confidence interval (95%) were used to evaluate the methods.

(3) Recovery, %= x 100

#### 3.6.5. Robustness

Small, deliberate changes to selected crucial parameters of the proposed method were conducted in order to assess the robustness of the HPLC method. Specifically, the concentration (18–20 mΜ) and the pH (2.7–2.9) of the phosphate buffer (mobile phase A), the column temperature (24–26 °C), the %Methanol at the beginning of the gradient elution (mobile phase B) (39.0 to 40% *v*/*v*) and the injection volume (19 to 21 μL) were investigated using the Plackett–Burman experimental design. The results were an evaluation based on peak response (area/IS area) and the resolution [34].

## 4. Conclusions

In the present study, a reliable HPLC fluorometric method was developed for the simultaneous quantitative and qualitative determination of five amino acids (L-leucine, L-isoleucine, L-histidine, L-valine, and β-alanine) encapsulated in chocolate pellets used as a dietary supplement. Pre-column derivatization with an OPA reagent was applied before the analysis for the fluorimetric detection of the analytes, whereas their chromatographic separation was performed using a C_18_ column and a mixture of the phosphate buffer (pH = 2.8; 20 mM)-methanol as mobile phase in gradient elution. For the purification of the samples and the quantitative recovery of amino acids from the substrate, three extraction techniques were developed and compared: solid-phase extraction, using two different SPE cartridges (NH_2_, Diol), and liquid–solid extraction (LSE). The optimization of both the derivatization parameters and the extraction method were evaluated using experimental design. The proposed method was validated according to ICH guidelines and demonstrated high precision, accuracy, and sensitivity. This procedure can be successfully applied for the determination of the five amino acids in chocolate pellet formulations (routine analysis) or to trace them on a chocolate substrate (trace analysis).

## Figures and Tables

**Table 1:** System suitability.

Parameters	Retention Time (min)	k’	Resolution	Tailing Factor
Histidine	6.45	-	-	1.217
Alanine	16.78	1.599	31.518	1.209
Valine	23.75	2.683	20.922	1.203
Isoleucine	27.60	3.280	9.486	1.193
Leucine	29.07	3.505	2.831	1.207
IS	30.65	3.749	2.695	1.198

**Table 2:** Accuracy—LSE.

Standard	C (Added) (μg mL^−1^)	Observed (ng mL^−1^)	Recovery (%)
		Mean ± SD	Mean ± SD
Histidine	0.09	0.09 ± 0.00	97.8 ± 0.2
	3.00	2.97 ± 0.01	99.0 ± 0.4
	5.00	4.92 ± 0.06	98.3 ± 1.1
Average (*n* = 9)			98.4 ± 0.9
Confidence interval (95%)			97.5–99.3
Alanine	0.02	0.02 ± 0.00	98.5 ± 0.5
	1.00	1.01 ± 0.01	100.6 ± 0.7
	2.00	2.00 ± 0.04	99.9 ± 1.2
Average (*n* = 9)			99.6 ± 1.7
Confidence interval (95%)			97.9–101.4
Valine	0.03	0.03 ± 0.00	99.1 ± 0.5
	5.00	4.92 ± 0.02	98.4 ± 0.4
	8.00	7.86 ± 0.01	98.2 ± 0.1
Average (*n* = 9)			98.6 ± 0.6
Confidence interval (95%)			98.0–99.2
Isoleucine	0.03	0.03 ± 0.00	98.3 ± 0.4
	5.00	4.94 ± 0.01	98.8 ± 0.1
	8.00	7.90 ± 0.04	98.8 ± 0.5
Average (*n* = 9)			98.6 ± 0.5
Confidence interval (95%)			98.2–99.1
Leucine	0.06	0.06 ± 0.00	99.1 ± 0.2
	10.00	10.05 ± 0.09	100.5 ± 0.9
	15.00	15.17 ± 0.11	101.1 ± 0.7
Average (*n* = 9)			100.2 ± 1.1
Confidence interval (95%)			99.1–101.4

**Table 3:** Accuracy—PE (Diol and NH_2_).

Standard	C (Added) (μg mL^−1^)	SPE/Diol		SPE/NH_2_	
		Observed (ng mL^−1^)	Recovery (%)	Observed (ng mL^−1^)	Recovery (%)
		Mean ± SD	Mean ± SD	Mean ± SD	Mean ± SD
Histidine	0.09	0.09 ± 0.00	98.5 ± 0.7	0.09 ± 0.00	96.1 ± 0.2
	3.00	2.93 ± 0.02	97.7 ± 0.5	2.92 ± 0.01	97.2 ± 0.2
	5.00	4.87 ± 0.01	97.4 ± 0.2	4.88 ± 0.01	97.5 ± 0.2
Average (*n* = 9)			97.8 ± 0.7	Average (*n* = 9)	96.9 ± 0.7
Confidence interval (95%)			97.1–98.6	Confidence interval (95%)	96.2–97.6
Alanine	0.02	0.02 ± 0.00	98.9 ± 0.1	0.02 ± 0.00	96.7 ± 0.4
	1.00	1.01 ± 0.01	100.9 ± 0.8	1.01 ± 0.01	101.3 ± 1.3
	2.00	2.03 ± 0.00	101.7 ± 0.2	2.06 ± 0.03	102.9 ± 1.3
Average (*n* = 9)			100.5 ± 1.3	Average (*n* = 9)	100.3 ± 3.0
Confidence interval (95%)			99.2–101.8	Confidence interval (95%)	97.3–103.3
Valine	0.03	0.03 ± 0.00	99.1 ± 0.3	0.03 ± 0.00	97.1 ± 0.2
	5.00	4.96 ± 0.03	99.2 ± 0.5	4.95 ± 0.03	99.1 ± 0.5
	8.00	7.99 ± 0.02	99.9 ± 0.2	8.03 ± 0.08	100.4 ± 0.9
Average (*n* = 9)			99.4 ± 0.5	Average (*n* = 9)	98.9 ± 1.6
Confidence interval (95%)			98.9–100.0	Confidence interval (95%)	97.3–100.4
Isoleucine	0.03	0.03 ± 0.00	98.0 ± 0.1	0.03 ± 0.00	97.0 ± 0.6
	5.00	4.90 ± 0.02	98.0 ± 0.5	4.85 ± 0.02	96.9 ± 0.3
	8.00	7.83 ± 0.05	97.9 ± 0.6	7.73 ± 0.02	96.7 ± 0.2
Average (*n* = 9)			98.0 ± 0.5	Average (*n* = 9)	96.9 ± 0.5
Confidence interval (95%)			97.5–98.4	Confidence interval (95%)	96.4–97.3
Leucine	0.06	0.06 ± 0.00	100.4 ± 1.3	0.06 ± 0.00	96.5 ± 0.4
	10.00	10.07 ± 0.08	100.7 ± 0.8	9.82 ± 0.04	98.2 ± 0.4
	15.00	15.10 ± 0.23	100.6 ± 1.5	14.85 ± 0.06	99.0 ± 0.4
Average (*n* = 9)			100.6 ± 1.3	Average (*n* = 9)	97.9 ± 1.2
Confidence interval (95%)			99.2–101.9	Confidence interval (95%)	96.7–99.1

**Table 4:** Final experimental conditions.

Parameter	Optimal Value	Selected Value
Chocolate weight (mg)	848.2	850.0
Centrifugation time (min)	18.3	20.0
Freezing time (min)	43.3	45.0
Ultrasonic time (min)	20.8	20.0
Initial volume of diluent (mL)	6.5	5.0
pH (initial) diluent	5.0	5.0
Volume of sample on SPE (mL)	2.2	2.0
Elution volume on SPE (μL)	300.0	300.0

**Table 5:** Accuracy—LSE/ SPE after experimental design.

Standard	C (Added) (ng mL^−1^)	Observed (ng mL^−1^)	Recovery (%)
		Mean ± SD	Mean ± SD
Histidine	20.0	18.7 ± 0.1	93.7 ± 0.6
	100.0	93.3 ± 0.4	93.3 ± 0.4
	500.0	460.2 ± 2.5	92.0 ± 0.5
Average (*n* = 9)			93.0 ± 0.9
Confidence interval (95%)			92.1–93.9
Alanine	20.0	18.2 ± 0.1	91.2 ± 0.4
	100.0	90.3 ± 0.6	90.3 ± 0.6
	500.0	446.2 ± 0.9	89.2 ± 0.2
Average (*n* = 9)			90.2 ± 1.0
Confidence interval (95%)			89.3–91.2
Valine	30.0	27.2 ± 0.3	90.8 ± 0.8
	200.0	179.9 ± 1.2	90.0 ± 0.6
	1000.0	885.0 ± 7.5	88.5 ± 0.7
Average (*n* = 9)			89.7 ± 1.3
Confidence interval (95%)			88.5–91.0
Isoleucine	100.0	95.1 ± 0.3	95.1 ± 0.3
	200.0	187.5 ± 0.4	93.7 ± 0.2
	1000.0	938.3 ± 0.9	93.8 ± 0.1
Average (*n* = 9)			94.2 ± 0.7
Confidence interval (95%)			93.5–94.9
Leucine	50.0	48.0 ± 0.1	96.0 ± 0.2
	200.0	191.5 ± 1.4	95.7 ± 0.7
	1000.0	949.7 ± 1.7	95.0 ± 0.2
Average (*n* = 9)			95.6 ± 0.7
Confidence interval (95%)			94.9–96.2

**Table 6:** Prediction error.

	%Recovery				
	Histidine	Alanine	Valine	Isoleucine	Leucine
**Predicted Value–LSE**	90.6	89.5	86.4	87.0	89.4
**Predicted Value–SPE**	87.1	84.0	84.8	81.2	86.6
**Predicted value (average)**	88.9	86.8	85.6	84.1	88.0
**Experimental value (average)**	93.0	90.2	89.7	94.2	95.6
**%Prediction Error**	4.4	3.8	4.6	10.7	7.9

**Table 7:** Results of linearity regression, correlation coefficient, LOD, and LOQ for amino acids.

Standard	Calibration Range (μg mL^−1^)	Regression Equation	r^2^	LOD (μg mL^−1^)	LOQ (μg mL^−1^)
Histidine	0.02–5.0	27.9x − 0.23	0.9992	0.01	0.02
Alanine	0.01–2.0	95.96x − 1.89	0.9991	0.002	0.01
Valine	0.01–8.0	18.67x − 0.06	0.9996	0.002	0.01
Isoleucine	0.01–8.0	18.96x − 0.13	0.9997	0.004	0.01
Leucine	0.03–15.0	10.15x − 0.04	0.9991	0.01	0.03

**Table 8:** Recommended daily dosage and composition of the formulation.

Amino Acids	Recommended Dosage (mg)/Day	Quantity (mg)/ Formulation
Leucine	1700	540
Isoleucine	850	270
Valine	850	270
Histidine	520	175
Alanine	750	250
Milk chocolate (excipient.)	-	4.6 g

## Supplementary Materials

The following are available online: Figure S1: Ideal values of the parameters studied during the optimization of the OPA-amino acids’ derivatization conditions. Figure S2: Pareto charts for the robustness test of HPLC instrumental parameters (ratio & resolution) for A) Histidine, B) Alanine, C) Valine, D) Isoleucine, E) Leucine. Table S1: Molecular structure, molecular weight, pKa and isoelectric point of amino acids, Table S2A: Design experiments and requirements used in the experimental design of LSE method, Table S2B: Conducted experiments for the optimization of LSE method, Table S3A: Design experiments and requirements used in the experimental design of SPE method, Table S3B: Conducted experiments for the optimization of SPE method, Table S4: Intermediate precision, Table S5: Conducted experiments with Box-Behnken design for the evaluation of the robustness.

## Sample Availability

Samples of the compounds are not available from the authors.
