# Supplementary material for: Fluorimetric Analysis of Five Amino Acids in Chocolate: Development and Validation
Source: Molecules. 2021 Jul 16;26(14):4325. doi: 10.3390/molecules26144325 (PMC8308014; doi:10.3390/molecules26144325)
Supplement: Supplementary file 1 [file molecules-26-04325-s001.zip › molecules-1297346-supplementary.pdf]

molecules

MDPI

Supplementary Material

Fluorimetric analysis of five amino acids in chocolate: Development and validation

Maria S. Synaridou [1], Vasilis Tsamis [1], Georgia Sidiropoulou [1], Constantinos K. Zacharis [1], Irene Panderi [2] and Catherine K. Markopoulou [1,*]

[1] Laboratory of Pharmaceutical Analysis, Department of Pharmaceutical Technology, School of Pharmacy, Aristotle University of Thessaloniki, 54124, Greece; msynarid@pharm.auth.gr (M.S.S); vasilistsm@gmail.com (V.T), sgeorgiae@gmail.com (G.S.); czacharis@pharm.auth.gr (C.K.Z.); amarkopo@pharm.auth.gr (C.K.M.)
[2] National and Kapodistrian University of Athens, Faculty of Pharmacy, Laboratory of Pharmaceutical Analysis, Panepistimiopolis, 15771, Athens, Greece; ipanderi@pharm.uoa.gr (I.P.)
* Correspondence: amarkopo@pharm.auth.gr (C.K.M); Tel.: +30 2310 997665

Table S1. Molecular structure, molecular weight, pKa and isoelectric point of amino acids.

Amino acids	Molecular structure	Molecular weight (g/mol)	pKa (-COOH)	pKa ($-NH_3^+$)	Isoelectric point (pI)
b-Alanine (Ala)	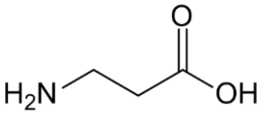	280.45	2.34	9.69	6.02
L-Histidine (His)	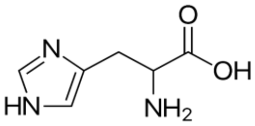	155.15	1.82	9.17	7.59
L-Valine (Val)	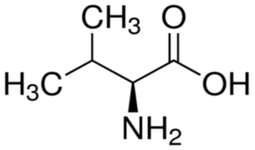	117.15	2.32	9.62	5.97
L-Leucine (Ile)	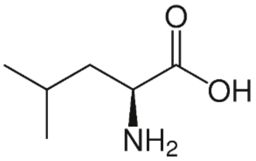	131.17	2.36	9.60	5.98
L-Isoleucine (Leu)	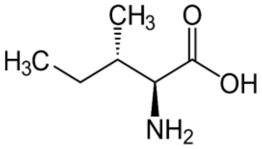	131.17	2.36	9.60	5.98

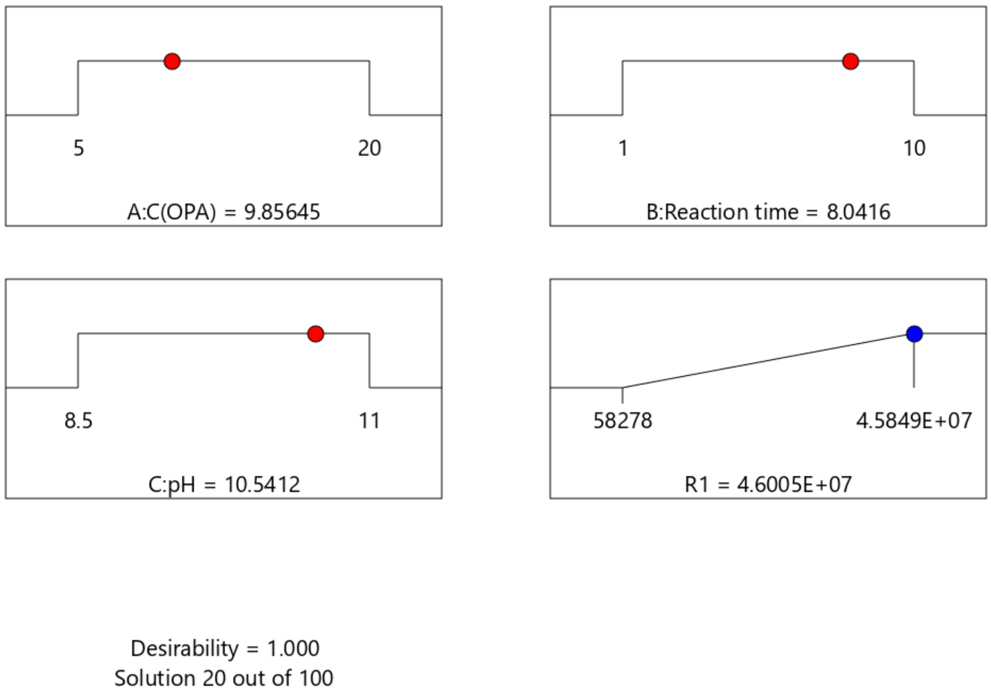

Figure S1. Ideal values of the parameters studied during the optimization of the OPA-amino acids' derivatization conditions.

Table S2. A: Design experiments and requirements used in the experimental design of LSE method.

Name	Goal	Lower Limit	Upper Limit	Lower Weight	Upper Weight	Importance
A: Chocolate weight	maximize	500	1500	1	1	5
B: Centrifugation time	minimize	5	30	1	1	3
C: Freezing time	minimize	30	90	1	1	3
D: Ultrasonic time	minimize	5	30	1	1	3
%Recovery Alanine	maximize	55	100	1	1	4
%Recovery Histidine	maximize	61	100	1	1	4
%Recovery Valine	maximize	55	99	1	1	4
%Recovery Isoleucine	maximize	50	99	1	1	3
%Recovery Leucine	maximize	60	100	1	1	3

Table S2. B: Conducted experiments for the optimization of LSE method.

**1st step: LSE – Experimental Design**					
**Std**	**Run**	**Factor 1 A: Chocolate weight (mg)**	**Factor 2 B: Centrifugation time (min)**	**Factor 3 C: Freezing time (min)**	**Factor 4 D: Ultrasonic time (min)**
10	**1**	1500	5	30	30
11	**2**	500	30	30	30
7	**3**	500	30	90	5
12	**4**	1500	30	30	30
14	**5**	1500	5	90	30
1	**6**	500	5	30	5
9	**7**	500	5	30	30

3	**8**	500	30	30	5
4	**9**	1500	30	30	5
2	**10**	1500	5	30	5
15	**11**	500	30	90	30
16	**12**	1500	30	90	30
6	**13**	1500	5	90	5
13	**14**	500	5	90	30
5	**15**	500	5	90	5
8	**16**	1500	30	90	5

Table S3. A: Design experiments and requirements used in the experimental design of SPE method.

Name	Goal	Lower Limit	Upper Limit	Lower Weight	Upper Weight	Importance
A: V (init.) diluent	minimize	5	7	1	1	3
B: pH (init.) diluent	is in range	1	5	1	1	3
C: V of sample on SPE	maximize	1	3	1	1	3
D: elution V on SPE	minimize	300	1500	1	1	3
% Recovery Alanine	maximize	49	101	1	1	3
% Recovery Histidine	maximize	48	99	1	1	3
% Recovery Valine	maximize	47	101	1	1	3
% Recovery Isoleucine	maximize	44	99	1	1	3
% Recovery Leucine	maximize	60	99	1	1	3

Table S3. B: Conducted experiments for the optimization of SPE method.

**2nd step: SPE – Experimental Design**					
**Std**	**Run**	**Factor 1 A: Initial volume of diluent (mL)**	**Factor 2 B: pH (initial) of diluent**	**Factor 3 C: Sample volume on SPE (mL)**	**Factor 4 D: Elution volume on SPE (μL)**
11	**1**	5	5	1	1500
3	**2**	5	5	1	300
7	**3**	5	5	3	300
6	**4**	15	1	3	300
12	**5**	15	5	1	1500
5	**6**	5	1	3	300
8	**7**	15	5	3	300
4	**8**	15	5	1	300
14	**9**	15	1	3	1500
16	**10**	15	5	3	1500
10	**11**	15	1	1	1500
15	**12**	5	5	3	1500
9	**13**	5	1	1	1500
1	**14**	5	1	1	300
13	**15**	5	1	3	1500
2	**16**	15	1	1	300

Table S4. Intermediate precision.

Standard	C(added) (μg mL^{-1})	%RSD		
		1st day	2nd day	3rd day
Histidine	0.02	0.91	1.68	1.75
	3.0	0.78	1.03	1.11
	5.0	0.39	0.80	0.97
Average (n = 9)		0.69	1.17	1.28
Alanine	0.01	0.85	0.96	1.56
	1.0	0.29	0.73	0.93
	2.0	0.33	0.41	0.91
Average (n = 9)		0.49	0.70	1.13
Valine	0.01	0.71	0.83	1.35
	5.0	0.59	0.80	1.10
	8.0	0.51	0.64	0.97
Average (n = 9)		0.60	0.76	1.14
Isoleucine	0.01	1.25	1.56	1.70
	5.0	1.03	1.22	1.51
	8.0	0.87	0.93	1.22
Average (n = 9)		1.05	1.24	1.48
Leucine	0.03	0.99	1.20	1.44
	10.0	0.93	1.23	1.76
	15.0	0.76	1.05	1.30
Average (n = 9)		0.89	1.16	1.50

Table S5. Conducted experiments with Plackett-Burman design for the evaluation of the robustness.

Design: 2**(7-4) design (Spreadsheet1)						
Standard Run	CenterPt	Buffer concentration (mM)	pH Buffer	Temperature (column) (°C)	Initial concentration of methanol (% v/v)	Injection volume (μL)
4	1	20.00000	2.900000	24.00000	40.00000	19.00000
8	1	20.00000	2.900000	26.00000	40.00000	21.00000
3	1	18.00000	2.900000	24.00000	39.00000	21.00000
6	1	20.00000	2.700000	26.00000	39.00000	21.00000
2	1	20.00000	2.700000	24.00000	39.00000	19.00000
9 (C)	0	19.00000	2.800000	25.00000	39.50000	20.00000
10 (C)	0	19.00000	2.800000	25.00000	39.50000	20.00000
1	1	18.00000	2.700000	24.00000	40.00000	21.00000
5	1	18.00000	2.700000	26.00000	40.00000	19.00000
11 (C)	0	19.00000	2.800000	25.00000	39.50000	20.00000
7	1	18.00000	2.900000	26.00000	39.00000	19.00000

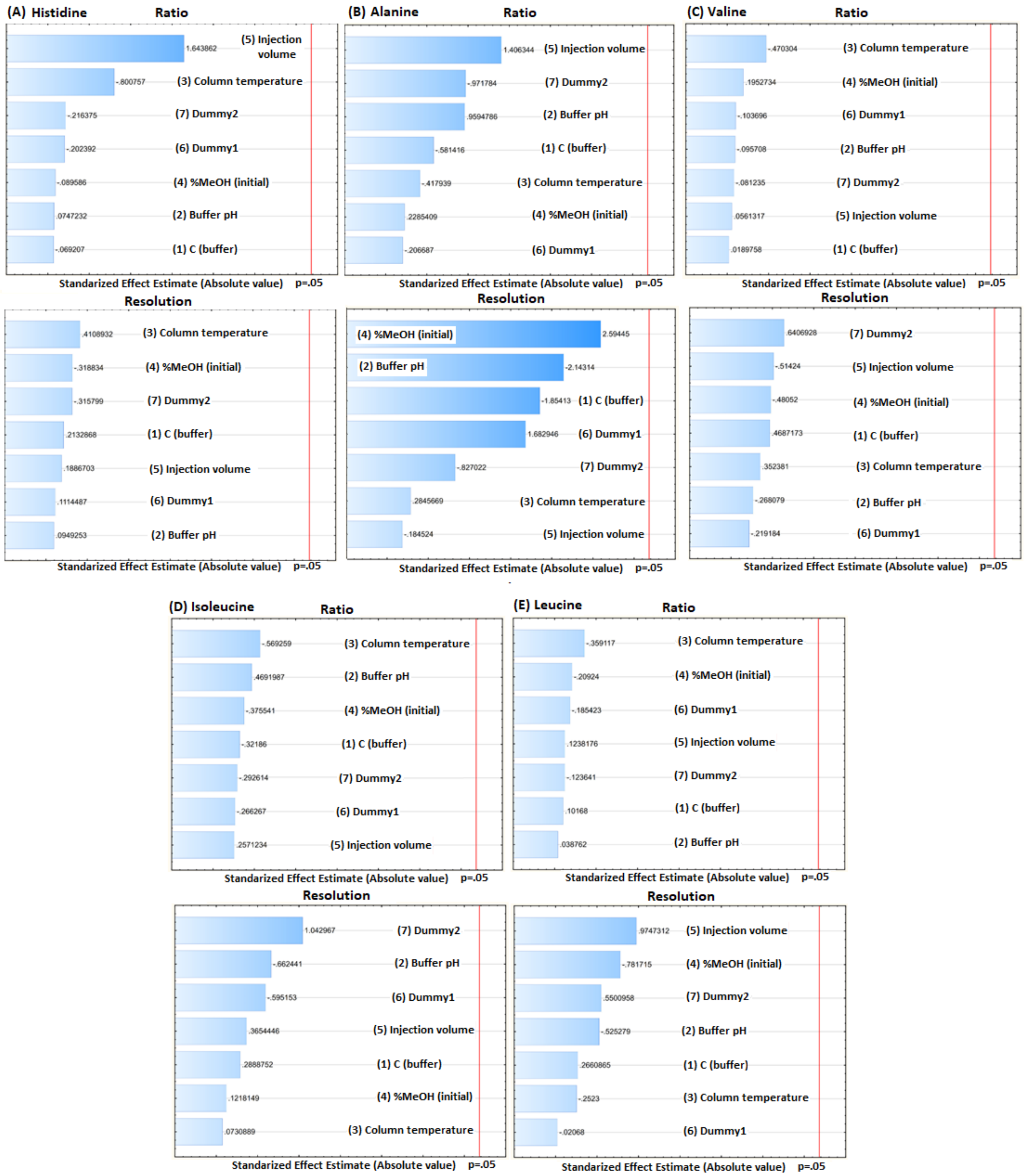

Figure S2. Pareto charts for the robustness test of HPLC instrumental parameters (ratio & resolution) for A) Histidine, B) Alanine, C) Valine, D) Isoleucine, E) Leucine.
